# Endophytic *Beauveria bassiana* increases galling of ‘Rutgers’ tomato roots with *Meloidogyne incognita*

**DOI:** 10.21307/jofnem-2021-072

**Published:** 2021-08-05

**Authors:** Shalini Yerukala, Ernest C. Bernard, Kimberly D. Gwinn, David M. Butler, Parwinder S. Grewal, Bonnie H. Ownley

**Affiliations:** 1 Department of Entomology and Plant Pathology, University of Tennessee, 370 Plant Biotechnology, 2505 E. J. Chapman Drive, Knoxville, TN; 2 Department of Plant Sciences, University of Tennessee, 2431 Joe Johnson Drive, Knoxville, TN; 3 Division of Research, Graduate Studies, and New Program Development, The University of Texas, Rio Grande Valley, 1201 West University Drive, Edinburg, TX

**Keywords:** *Beauveria bassiana*, Biological control, Egg hatch, Endophyte, Juvenile survival, *Meloidogyne incognita*, Root galling, Root knot nematode, *Solanum lycopersicum*, Tomato

## Abstract

*Beauveria bassiana* is endophytic in many plant species and has been shown to protect host plants against insect pests and plant pathogens. However, less is known about its activity against plant-parasitic nematodes. In vitro and plant assays were conducted to determine the effect of *B. bassiana* 11-98 (Bb) on *Meloidogyne incognita* (root-knot nematode; RKN). *Beauveria bassiana* was confirmed as an endophyte in ‘Rutgers’ tomato and colonization patterns of Bb in ‘Rutgers’ (highly susceptible to RKN) were compared with those in ‘Mountain Spring’ (less susceptible to RKN). In greenhouse tests with ‘Rutgers’ at 30 and 60 days after treatment (DAT) with RKN and Bb, there were few differences in plant growth variables among treatments in repeated trials. However, RKN root galling and egg count/root system were enhanced in plants treated with Bb at 60 DAT. In an in vitro assay with egg masses from greenhouse tests, the percentages of hatched eggs, and mobile and immobile nematodes did not differ significantly for RKN and RKN+Bb treatments. The presence of viable Bb from roots was confirmed by collecting egg suspensions from root galls and plating them on selective medium. Colonies of Bb were verified on agar medium, but no parasitism of RKN eggs was observed. Research is needed to investigate factors responsible for increased galling by RKN in the presence of endophytic Bb in ‘Rutgers’ tomato.

More than 4,100 species of plant-parasitic nematodes have been identified (Decraemer and Hunt, 2006) and worldwide economic losses due to crop damage have been estimated at $80 to $118 billion dollars per year (Nicol et al., 2011; Sasser and Freckman, 1987). Plant parasitic nematodes that feed on plant roots cause direct damage by reducing nutrient and water flow (Bernard et al., 2017; Nicol et al., 2011; Sasser and Freckman, 1987; Schouteden et al., 2015), and indirect damage by providing entry wounds for secondary pathogens (Shalini et al., 2014). Of all identified nematodes, only 15% cause significant economic crop losses (Bernard et al., 2017; Koenning et al., 1999). Genera of highest economic importance in the U.S. include *Meloidogyne*, *Heterodera*, *Pratylenchus*, *Hoplolaimus*, *Xiphinema*, and *Rotylenchulus* (Bernard et al., 2017; Koenning et al., 1999).

*Meloidogyne incognita*, the southern root-knot nematode (RKN), is the most devastating threat to agricultural crop production worldwide (Postnikova et al., 2015) and is a major threat to global food security (Bernard et al., 2017). Root knot nematode is sedentary and establishes a permanent feeding site within the plant host root to obtain nutrients and complete its lifecycle (Bernard et al., 2017; Koenning et al., 1999). Moreover, RKN is cosmopolitan in distribution, has a wide host range (Bernard et al., 2017), and can cause plant disease complexes with fungi and bacteria (Shalini et al., 2014).

Chemical nematicides are often used to control plant parasitic nematodes, but some are highly toxic and can cause significant environmental pesticide pollution (Cáceres and Venkateswarlu, 2018; Gamboa et al., 2020; Mishra et al., 2020; Vandergragt et al., 2019). Alternative, sustainable, ecofriendly management strategies to control plant-parasitic nematodes and reduce the pollution impact of nematicides are needed. Identification and implementation of endophyte-host resistance toward plant-parasitic nematodes is a potential control strategy (Bernard et al., 2017).

The fungal entomopathogen *Beauveria bassiana* (Balsamo) Vuillemin belongs to the phylum Ascomycota: Hypocreales and is routinely used for control of insects that cause damage in protected agriculture systems. The fungus increases mortality in all life stages of insects including eggs (Long et al., 1998; Lord, 2009; Lynch and Lewis, 1978; Marannino et al., 2006), and arachnid pests (Kaaya and Hassan, 2000; Shi and Feng, 2004).

*Beauveria bassiana* (Bb) has been reported as an endophyte of a large number of diverse plants, including food and fiber crop species (Jaber and Ownley, 2018).

Endophytic colonization of gramineous crops by Bb has been reported for maize (Bing and Lewis, 1991; Gurulingappa et al., 2010), sorghum (Tefera and Vidal, 2009), and wheat (Gurulingappa et al., 2010; Russo et al., 2015). Endophytic colonization by Bb has been reported for several leguminous crops including common or snap bean (Akutse et al., 2013; Gurulingappa et al., 2010; Ownley et al., 2008; Parsa et al., 2013), cowpea (Maketon et al., 2013), fava bean (Akutse et al., 2013), and soybean (Russo et al., 2015). Endophyte colonization of solanaceous crops by Bb has been documented in potato, tomato (Gurulingappa et al., 2010; Leckie, 2002; Ownley et al., 2008; Resquín-Romero et al., 2016), and tobacco (Russo et al., 2015), as well as solanaceous jimsonweed (Jones, 1994). Endophyte colonization of two fiber crops, cotton (Gurulingappa et al., 2010; Jones, 1994; Ownley et al., 2008) and jute (Biswas et al., 2012) has been reported. Trees such as American hornbeam (Bills and Polishook, 1991), cocoa (Evans et al., 2003; Posada and Vega, 2005), date palm (Arab and El-Deeb, 2012; Gómez-Vidal et al., 2006), pecan (Ramakuwela et al., 2020), and pine (Brownbridge et al., 2012; Ganley and Newcombe, 2006; Lefort et al., 2016) are colonized by Bb endophytes. Reports of Bb colonization of banana, opium poppy (Quesada-Moraga et al., 2006) and coffee (Posada and Vega, 2006; Posada et al., 2007) have been published, as have reports on pumpkins (Gurulingappa et al., 2010), and the weed species, cocklebur (Jones, 1994).

Endophytic colonization of plants by Bb have increased tolerance to insect pests (Rondot and Reineke, 2013), and provided season-long protection against tunneling insects (Arab and El-Deeb, 2012), such as stem borers (Cherry et al., 2004), and poppy stem gall wasp (Quesada-Moraga et al., 2009); sucking insects (Gurulingappa et al., 2011), such as mealybug, grape leafhopper (Rondot, and Reineke, 2018), and aphids (Lopez et al., 2014; Maketon et al., 2013); chewing insects (Akello et al., 2008), such as beetles (Kreutz et al., 2004) and grasshoppers (Pelizza et al., 2017); and others, including fire ants (Broome et al., 1976).

As an endophyte, Bb can protect host plants against insect pests (Rondot and Reineke, 2013), and plant pathogens (Gómez-Vidal et al., 2006; Jaber and Salem, 2014; Ownley et al., 2010). Isolate Bb 11-98 was significantly more toxic to cotton bollworm (*Helicoverpa zea*) in diet tests than other strains evaluated (Leckie et al., 2008), and has been recovered from leaf, stem, and root tissues of 18-week-old tomato plants that were initially seed-inoculated with Bb (Powell et al., 2009). Tomato seedlings colonized with Bb 11-98 were more tolerant of soilborne fungal and oomycete pathogens, i.e., *Rhizoctonia* (Bishop, 1999) and *Pythium* (Clark, 2006), and Bb 11-98 applied to seed protected cotton foliage against a bacterial pathogen (Griffin, 2007). In addition, Jaber and Salem (2014) reported that squash plants colonized by *B. bassiana* were protected from *Zucchini yellow mosaic virus*. There are likely multiple mechanisms for disease suppression with endophytic *B. bassiana*, including stimulation of plant defenses through induced systemic resistance (Dara, 2019; Jaber and Ownley, 2018).

In contrast, little is known about the effectiveness of endophytic Bb against plant-parasitic nematodes. Endophytic colonization of hosts offers potential for developing sustainable plant protection strategies utilizing an entomopathogenic fungus for management of nematode diseases (Liu et al., 2008; Zhao et al., 2013). *Epichloe* endophytes of grasses provide protection against RKN (Jia et al., 2013; Kimmons et al., 1990; Meyer et al., 2013). A cultivar of tall fescue, *Festuca arundinacea* (MaxQ), infected with a strain of *E. coenophiala* that does not produce the mammalian toxin ergovaline, reduced nematode penetration, and second-stage juveniles (J2) failed to complete their life cycle. In addition, root exudates were nematotoxic and inhibited egg hatch (Meyer et al., 2013).

Here, the potential of reducing egg hatch and root galling of RKN on tomato was investigated with in vitro and greenhouse assays. An isolate of Bb (11-98) shown to be an endophyte and to reduce plant disease in ‘Mountain Spring’ tomato was used (Bishop, 1999; Ownley et al., 2004, 2008; Seth, 2001). The specific objectives of the current study were to (i) determine and compare the ability of Bb 11-98 to endophytically colonize two tomato cultivars, Rutgers and Mountain Spring; and (ii) determine the impact of endophytic Bb in tomato roots on RKN pathogenesis as measured by root and shoot growth, fruit number, root galling, egg number, and mobility of hatched juveniles in greenhouse experiments.

## Materials and methods

### Endophytic Bb and culture media

Endophytic, entomopathogenic Bb isolate 11-98 (Dr. B. H. Ownley, University of Tennessee, Knoxville) was used for all experiments. The fungus was grown on Sabouraud dextrose agar (SDA) (Difco, Sparks, MD) or *Beauveria* selective medium (BSM; Doberski and Tribe, 1980). Endophytic status was confirmed by isolating the fungus from plant tissue on BSM. The medium was prepared with glucose (32 g), neopeptone (8 g), agar (12 g), and crystal violet (0.008 g) in 800 ml of deionized water. The medium was autoclaved for 45 min, 121°C, 15 psi, and cooled to ~55°C, after which cycloheximide (0.2 g/4 ml) and chloramphenicol (0.4 g/4 ml) were added before pouring the plates. The fungus was grown on SDA, prepared according to manufacturer’s instructions, and incubated at 25°C for approximately 3 to 4 weeks for production of conidia. Dry conidia were harvested from the surface of SDA culture plates with a camel-hair brush, and stored in glass vials in a desiccator and refrigerated (~4°C).

### Tomato cultivars

Tomato seeds were purchased from Park Seed (Greenwood, SC). Seed germination rate for each lot of seed was 85% for ‘Rutgers’ and 88% for ‘Mountain Spring’.

### Meloidogyne incognita

A culture of RKN used in all experiments was obtained from Dr. E. Bernard (University of Tennessee, Knoxville), and maintained on ‘Rutgers’ tomato plants in the greenhouse. This isolate is *M. incognita* Race 3 and was originally collected from an ornamental okra plant at the West Tennessee Research and Education Center, Jackson, TN.

### Seed treatment with Bb 11-98

A gnotobiotic assay was performed to confirm the endophytic ability of *B. bassiana* 11-98 in Rutgers and Mountain Spring tomato cultivars. Seeds (‘Rutgers’ and ‘Mountain Spring’ tomato) were coated with Bb 11-98 based on a ratio of 2 g seed: 1 ml 2% methyl cellulose solution containing 0.02% Tween 20 (USB, Cleveland, OH) and Bb (1 g dry conidia). Seeds were stirred until the coating of conidia appeared uniform and had started to dry. Coated seeds were spread on aluminum foil and air-dried in a biological safety cabinet for approximately 3 h. Dry, coated seeds were placed in a glass vial for storage at 4°C with a desiccant until needed. A replicated sample of treated seed was used to determine the density of Bb conidia per seed by standard dilution plating; 10 seeds were used per cultivar. Dilutions were plated onto BSM. The number of conidia per seed was log 7. The experiment was replicated and repeated once.

Culture tubes (24-mm outside diameter and 15-cm length) were filled with 20 cm^3^ vermiculite (Palmetto Vermiculite Co., Medium A-2, Woodruff, SC) and 20 ml of deionized water. Tubes were sealed with plastic caps, sterilized by autoclaving for 1 h on each of 2 consecutive days, cooled, and then transferred to a biosafety cabinet. In each culture tube, one seed was placed approximately 0.5 cm below the surface of the vermiculite for each treatment/cultivar combination. Tubes were recapped and placed in a growth chamber (Baxter Scientific Products, Deerfield, IL) with continuous light for 72 hr at 25°C. The tubes were then transferred to a walk-in growth chamber (Environmental Growth Chamber, Chagrin Falls, OH) at 25°C with an 18/6 hr light-dark regimen. Planted seeds were maintained for 21 days and percent germination was recorded.

### Isolation of Bb from tomato seedlings

Endophyte presence was determined in seedlings at the two true-leaf stage (21 days after planting). Seven seedlings of each treatment/cultivar combination were randomly selected from each trial and removed from tubes. Roots were rinsed free of vermiculite, and each plant was wrapped in a moistened paper towel and labeled. Each seedling was surface-sterilized with 95% ethanol for 1 min, followed by a 10% aqueous solution of commercial bleach (a.i., 6.0% sodium hypochlorite) for 1 min, and sterile deionized water for 1 min to remove excess bleach. Surface-sterilized seedlings were then placed on sterile paper towels to remove excess moisture.

Surface-sterilized tomato seedlings were aseptically cut into 1-cm sections (leaf, stem, and root) and placed on BSM. All sections of the surface-sterilized seedlings were plated onto BSM with five sections placed equidistant from one another per plate. BSM plates were sealed with Parafilm and incubated in darkness at 25°C. Plates were observed daily for emergence of the endophytic fungus from the cut edges of plant sections. Plant pieces exhibiting presence of a fungal endophyte were transferred to new plates containing fresh BSM. Presence or absence of fungal growth from the cut edges of leaf, stem or root tissue was recorded for each plant section and colonies were examined microscopically for conidiophore and conidial characteristics. No fungal hyphae emerged from cut sections of surface-sterilized control plants. Percent colonization of plants was calculated according to the formula developed by Akutse et al. (2013), where

$$Percent\;colonization\;=\left(\frac{Number\;of\;sections\;exhibiting\;fungal\;growth}{Total\;number\;of\;pieces\;plated}\right)\times 100$$

In addition to microscopic observation of the fungus, identification of Bb was confirmed with a cultivation-independent method. Genomic DNA was extracted from 7-day-old cultures of the fungus isolated from tomato seedlings in the gnotobiotic assay using Phire Plant Direct PCR Master Mix (Thermo Fisher Scientific, Waltham, MA) and ITS primers 1 and 2. The presence of a PCR product was confirmed with gel electrophoresis and SYBR Safe DNA gel stain (Thermo Fisher Scientific). For sequencing, PCR amplicons were cleaned of excess primer and unincorporated nucleotides with ExoSAP-IT (Thermo Fisher Scientific) following the manufacturer’s directions. Samples were sequenced at the University of Tennessee, Molecular Biology Resource Facility. Sequences obtained were submitted to the NCBI Nucleotide Blast alignment tool and identification of Bb was verified by comparisons with known Bb sequences in the nucleotide database.

The gnotobiotic assay was arranged in a completely randomized design in a growth chamber, with 17 replicate seeds for Trial 1 and 14 replicate seeds for Trial 2 of each treatment for each tomato cultivar. For analysis of colonization data, the study was a 2 × 3 factorial with two cultivars and three tissue types. Seedlings were selected randomly for assessment of colonization by Bb, with six seedlings in Trial 1 and seven seedlings in Trial 2. Data are presented as percentages, but the proportion values were transformed with arcsine of the square root of proportion values to satisfy assumptions of normality and equal variance, and analyzed for significance with a mixed model ANOVA (SAS 9.4). Least significant difference (LSD) was used to determine significant mean differences (*p* ≤ 0.05).

### Impact of endophytic Bb in tomato roots on RKN pathogenesis in greenhouse assay

The greenhouse experiment consisted of four treatments: (i) Bb seed treatment alone, (ii) Bb seed treatment + RKN, (iii) RKN alone, and (iv) Control (no Bb seed treatment, no RKN). ‘Rutgers’ tomato seeds (30 seeds per treatment) were surface-sterilized with 95% ethanol (1 min), followed by 10% bleach (Clorox) (1 min) and sterile deionized water (1 min), and placed on sterile paper towels to remove excess moisture. For treatments with Bb, seeds were treated with Bb in 2% methyl cellulose as described previously to achieve log 7 conidia per seed. Colony counts on seed were confirmed on BSM. Treatments without Bb were coated with the methyl cellulose sticker.

Seeds were treated as described for the gnotobiotic assay. Endophyte-treated tomato seeds and control seeds were sown in plug trays with potting mix (Sunshine #1 Natural & Organic, Sun Gro Horticulture, Agawan, MA). The trays were covered with a polyethylene cover until the seeds germinated. Germination rate was recorded daily, and seedlings were watered as needed.

Two weeks after planting Trial 1, seedlings were fertilized with 100 ml fish fertilizer (half-strength Alaska Fish Emulsion Fertilizer Concentrate 5-1-1, Pennington, Madison, GA). For Trial 2, 100 ml fish fertilizer was applied twice (2 weeks after planting and 1 week later). At 3 (Trial 1) or 4 (Trial 2) weeks after sowing, seedlings were transferred into individual pots (3.78-L), filled with sand (All Purpose Sand, Quikrete, Atlanta, GA) and potting soil (Sunshine #1 Natural & Organic) at a ratio of 2:1. Fertilizer (5 cm^3^ Osmocote Smart-Release Plant Food Plus Outdoor and Indoor, Scotts Company, Marysville, OH) was added to each pot near the root zone of the seedling at transplant. Fish fertilizer (100 ml) was applied at 1 week and 2 weeks after seedling transplant. Osmocote (5 cm^3^) was applied at 2 weeks after transplant also.

Five weeks after sowing seed, 10,000 RKN eggs in an aqueous suspension were dispensed into the root zone of each seedling by creating four holes around the root zone and adding 2,500 RKN eggs per hole. Control plants received no RKN. In addition, pots that received Bb as a seed treatment were drenched with 100 ml of Bb conidial suspension near the root zone of each plant 1 to 2 days before RKN inoculation to increase opportunity for root colonization and establishment of Bb in the rhizosphere. Concentrations of Bb in the drench solution were log 8/ml for Trial 1 and log 7/ml for Trial 2. The drench step was added to help ensure that Bb was established as an endophyte.

Thirty days after RKN inoculation, soil was removed from root systems of half of the plants and galls/root system were rated based on a standard rating scale of 1 to 10 (Zeck, 1971). Growth parameters, including shoot height (cm), shoot fresh weight (g), and root fresh weight (g) were measured. Sixty days after nematode inoculation, the remaining root systems were harvested, and the number of galls/root system were rated. Shoot height (cm), shoot fresh weight (g), root fresh weight (g), fruit weight (g), fruit number, and flower number were assessed.

Egg count was determined for all nematode treatments (RKN and RKN + Bb) for 60-day plants only (Hussey and Barker, 1973). Extracted eggs were counted by pouring 1 ml of egg suspension into a counting dish. Two 1-ml samples were assessed from each egg suspension per plant and counts were averaged. The egg suspensions (500 µl) extracted from greenhouse treatments RKN and RKN + Bb were inoculated onto BSM. The presence or absence of endophytic Bb growth from egg suspensions was observed and recorded.

The greenhouse experiment was designed as a randomized complete block. Treatments were replicated 10 times for Trial 1 and 6 times for Trial 2. Data from the two trials was pooled, transformed as needed, and analyzed for significance with a mixed model ANOVA (SAS 9.4). Least significant difference (LSD) was used to determine significant mean differences (*p* ≤ 0.05).

### In vitro assay with egg suspensions from greenhouse plants

For Greenhouse Trial 1, there were 10 replicate plants. At 30 days after RKN treatment, 5 plants were sacrificed for the 30-day data, leaving 5 plants for the 60-day data. For the in vitro data, egg masses were extracted from each RKN and RKN + Bb treatment replicate. These were incubated for 6 days, and 6 subsamples of each replicate were counted daily, and averaged for a replicate mean. For Greenhouse Trial 2, there were six replicate plants, which were split for the 30-day and 60-day data, leaving three replicate plants for the 60-day data and extraction of egg masses for the in vitro assay. Counts of hatched eggs and mobile and immobile nematodes were observed in four subsamples from each replicate daily, and averaged for a replicate mean, over a period of 10 days. Egg masses from the two trials were observed over more days in Trial 2 because of a slower egg hatch rate. The assays were terminated when the RKN treatment reached approximately 40% egg hatch.

Eggs were extracted from greenhouse treatments that included RKN (i.e., RKN and RKN + Bb). Egg hatch rate was determined in an in vitro assay. No additional Bb 11-98 was added. To evaluate the hatch rate from eggs extracted from greenhouse nematode treatments, samples were placed in a 96-well plate. Each well received 100 µl of egg suspension. The absolute number of RKN eggs present in each treatment replicate varied because eggs were taken directly from plant root systems in the greenhouse experiment. Data were collected on number and percentages of hatched and unhatched eggs, and immobile and mobile RKN.

Data are presented as percentages, but the proportion values were transformed with arcsine of the square root of proportion values to satisfy assumptions of normality and equal variance, and analyzed for significance with a mixed model ANOVA (SAS 9.4). Least significant difference (LSD) was used to determine significant mean differences (*p* ≤ 0.05).

## Results

### Ability of Bb 11-98 to endophytically colonize tomato

The colonization assay was conducted to confirm that Bb 11-98 was endophytic in ‘Rutgers’ tomato, and to compare the extent of colonization with ‘Mountain Spring’, which has been used in multiple studies with endophytic Bb. ‘Mountain Spring’ is less susceptible to RKN, while ‘Rutgers’ is very susceptible and is commonly used as a stock host for agronomically important *Meloidogyne* species.

**Figure 1:**
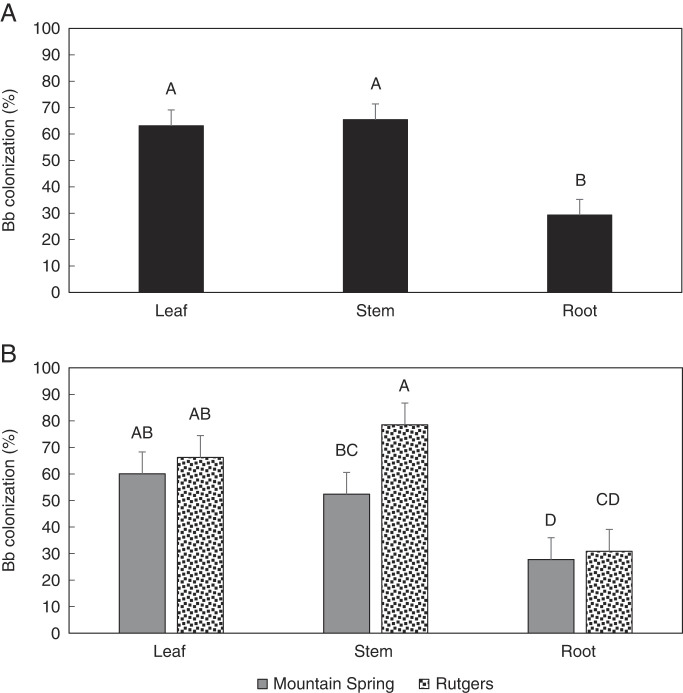
Percent colonization by endophytic *B. bassiana* (Bb) of leaf, stem, and root samples of tomato seedlings of ‘Mountain Spring’ and ‘Rutgers’ grown from Bb-treated seed. Data from two trials were pooled. Means for the control (no Bb treatment) = 0% colonization and are not shown. Bars with the same letter are not different according to an F-protected LSD at *p* ≤ 0.05. (A) Data for the two cultivars were combined: (B) data for the interaction of cultivar and plant sample.

At 10 to 15 days after plating seedling samples on BSM, white mycelial growth was observed from the cut edges of sections of leaf, stem, and roots of seedlings grown from Bb-coated seeds of both cultivars, while no fungal growth was observed in control plants, without endophyte treatment. Across both cultivars, there were significant differences in colonization of root, leaf, and stem samples (*p* < 0.0001). The colonization percentage by Bb was higher in stems and leaves from Bb treatments than in roots (Fig. 1A). Across all three sample types, there was a trend for greater colonization in ‘Rutgers’ than in ‘Mountain Spring’, but the difference was not significant (*p* = 0.0566), with 59 and 47%, respectively. Colonization was equally high for leaf samples (66 vs. 60%) and equally low for root samples (31 vs. 28%) for ‘Rutgers’ and ‘Mountain Spring’, respectively (Fig. 1B). However, percentage colonization of stem samples was higher (*p* = 0.05) in ‘Rutgers’ (79%), than in ‘Mountain Spring’ (52%) (Fig. 1B). Because colonization patterns were similar for the two cultivars, there was no significant interaction between cultivar and plant tissue type (*p* = 0.2350).

Identity of the fungus growing from leaf, stem and root samples was confirmed by culturing isolates on BSM in pure culture, followed by microscopic observations of conidia and conidiophores, as well as observations on cultural characteristics. Molecular identification was performed on isolates obtained in Trial 1. The fungus cultured from plant tissue had 100% ITS region sequence identity with isolates of *B. bassiana* in the NCBI nucleotide database.

### Seed germination

Seed germination rate was recorded for all treatments. In Trial 1, for seed treated with Bb, germination was 94% for ‘Mountain Spring’ and 82% for ‘Rutgers’. For control seed coated with methyl cellulose, germination was 82% for ‘Rutgers’ and 65% for ‘Mountain Spring’. In Trial 2, the germination rate was 86% in Bb-treated seed for ‘Rutgers’ and 96% for ‘Mountain Spring’. For methyl cellulose control seed, the germination rate was 96% in ‘Rutgers’ and 76% in ‘Mountain Spring’.

### Impact of endophytic Bb in tomato roots on RKN pathogenesis in greenhouse assay

The germination rate of ‘Rutgers’ was 92% for Bb-treated and control seed in Trial 1, while in Trial 2, germination was 100% for the methyl cellulose control and 76% in Bb-treated seed. The population of Bb on ‘Rutgers’ seed was log 7 for both trials.

**Figure 2:**
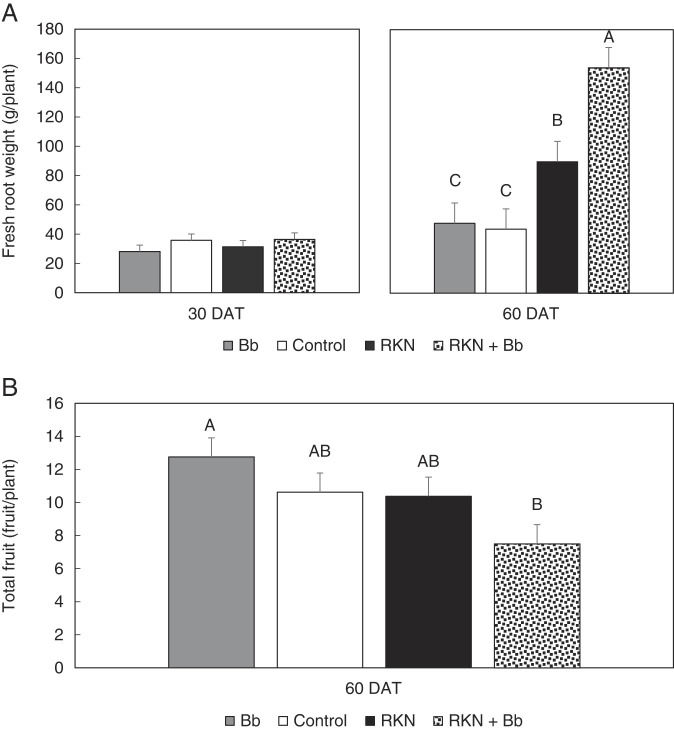
Effect of endophytic *B. bassiana* (Bb) and root-knot nematode (RKN) on (A) root fresh weight (g) and (B) fruit number of ‘Rutgers’ tomato evaluated in a greenhouse assay. Data for two trials were pooled for analysis. Plants were evaluated at 30 days and 60 days after treatment (DAT). Bars with the same letters or no letters are not different according to an F-protected LSD at *p* ≤ 0.05 (A) and *p* = 0.07 (B). Combined analyses were conducted for each incubation period and trial. Treatments were Bb, RKN, RKN + Bb, and Control.

In the greenhouse assay, growth of tomato plants treated with Bb + RKN were compared with three controls: Bb only, RKN only, and Control (no treatment). At 30 and 60 days after treatment (DAT), there were no differences among treatments for shoot height and fresh weight (data not shown). Similarly, at 30 DAT there were no differences in root fresh weight, but at 60 DAT, root fresh weight (*p* < 0.0001) was greater with the RKN + Bb treatment, intermediate in the RKN only, and least in the control and Bb only (Fig. 2A). Fruit weight was measured at 60 DAT, and there were no differences among treatments (data not shown). But fruit number trended higher (*p* = 0.07) in Bb only than in RKN + Bb (Fig. 2B). Flower number did not differ in either trial (data not shown).

**Figure 3:**
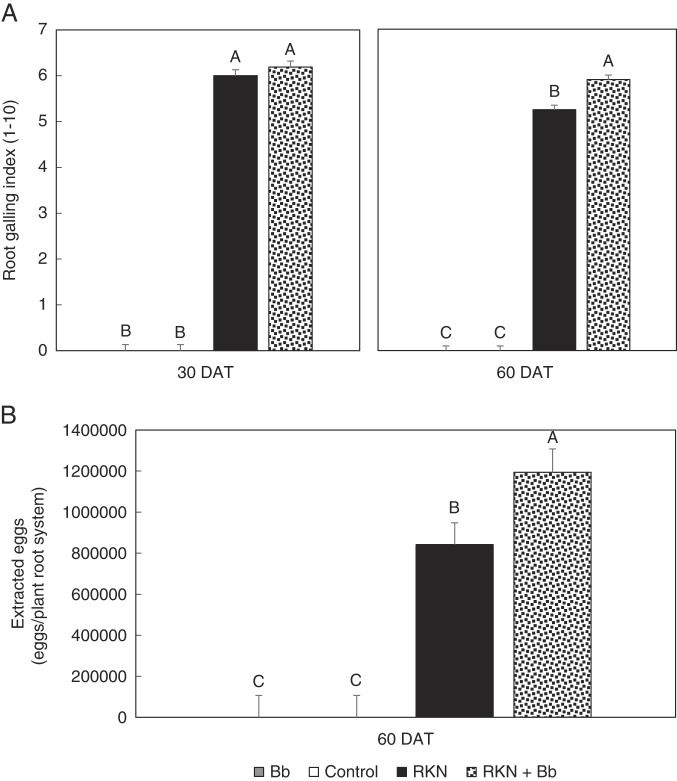
Effect of endophytic *B. bassiana* (Bb) and root-knot nematode (RKN) on (A) root galling index at 30 and 60 days after treatment (DAT) and (B) egg count number at 60 DAT of ‘Rutgers’ tomato evaluated in a greenhouse assay. Plants were evaluated in two trials, and data were pooled for analysis. Treatments were Bb, RKN, RKN+Bb, and Control. No galling or RKN eggs were observed in Bb only and the Control. Bars with the same letters are not different according to an F-protected LSD at *p* ≤ 0.05.

Root galling was similar with RKN only and RKN + Bb at 30 DAT; however, at 60 DAT, galling was significantly greater in the RKN + Bb treatment than with RKN alone (*p* < 0.0001) (Fig. 3A). No galls were found on plants that did not receive RKN (i.e., control and Bb only) (Fig. 3A). There were significant differences in egg count per root system among treatments (*p* < 0.0001) at 60 DAT (Fig. 3B). The egg count from RKN + Bb was significantly higher than RKN alone. There were no egg counts for control and Bb only.

### In vitro egg hatch assay from greenhouse plants

**Figure 4:**
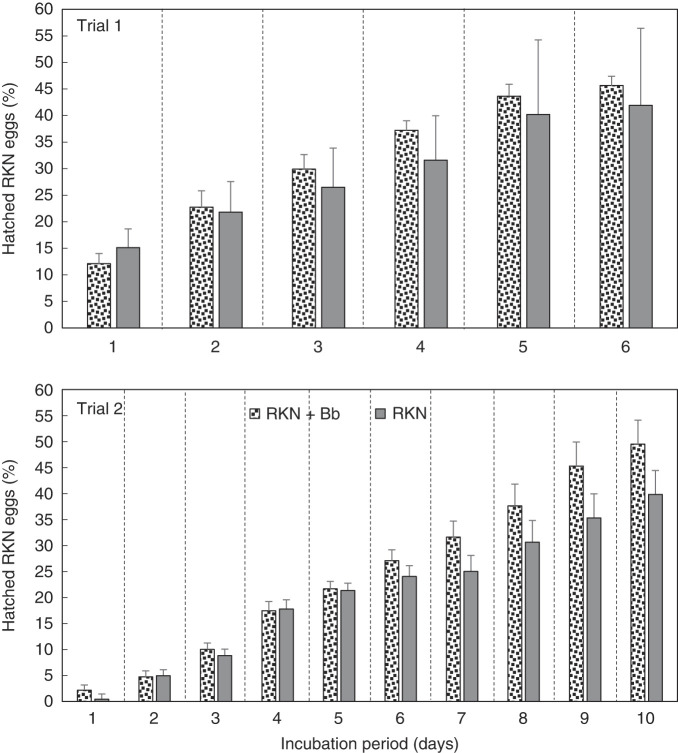
Effect of endophytic *B. bassiana* (Bb) on percentage egg hatch of root-knot nematode (RKN) extracted from ‘Rutgers’ tomato roots in a greenhouse assay. Treatments were RKN and RKN + Bb. Data for two trials is presented. For each trial, a separate analysis was performed for each day. Bars with no letters are not different according to an F-protected LSD at *p* ≤ 0.05.

**Figure 5:**
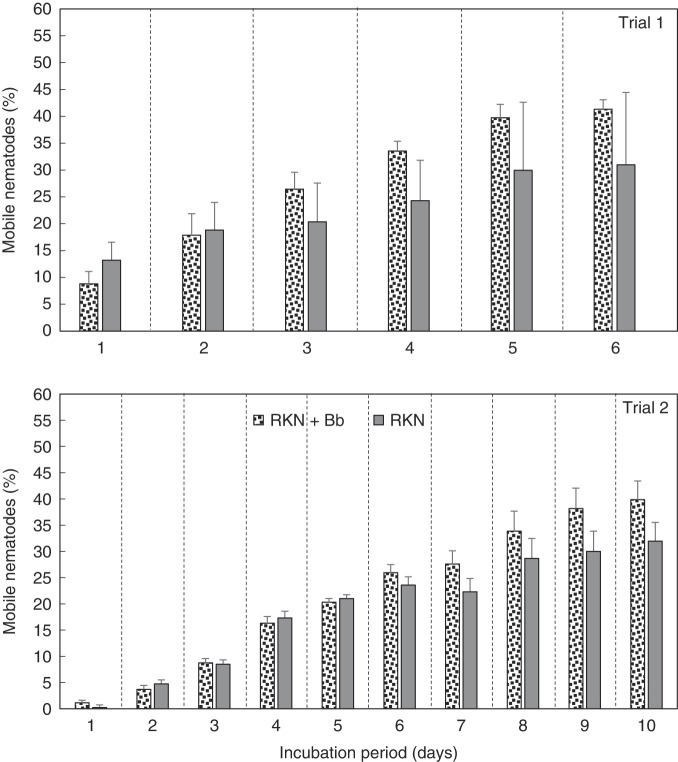
(A) Effect of endophytic *B. bassiana* (Bb) on percentage mobile root-knot nematodes (RKN) extracted from ‘Rutgers’ tomato roots in a greenhouse assay. Treatments were RKN and RKN + Bb. Data for two trials is presented. For each trial, a separate analysis was performed for each day. Bars with no letters are not different according to an F-protected LSD at *p* ≤ 0.05.

**Figure 6:**
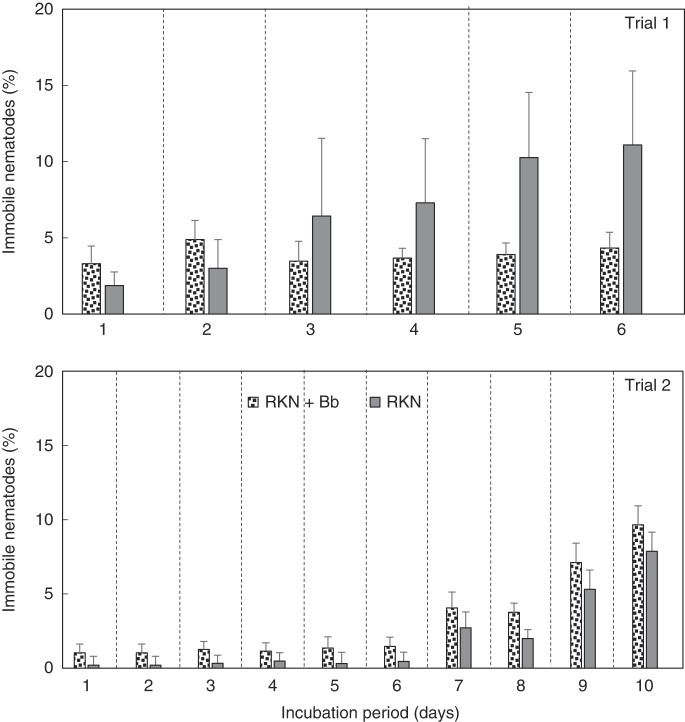
(A) Effect of endophytic *B. bassiana* (Bb) on percentage immobile root-knot nematodes (RKN) extracted from ‘Rutgers’ tomato roots in a greenhouse assay. Treatments were RKN and RKN + Bb. Data for two trials is presented. For each trial, a separate analysis was performed for each day. Bars with no letters are not different according to an F-protected LSD at *p* ≤ 0.05.

When egg hatch of Bb colonized plants was monitored, over the 6 days of Trial 1, and 10 days of Trial 2, percentage egg hatch increased similarly over the incubation period in both treatments (Fig. 4). In both trials, there was a trend for higher percentage egg hatch for RKN + Bb treatment than RKN alone; however, the difference was not significant. Overall, there was an increase in percentage mobile nematodes over time in both trials (Fig. 5). Similar to percentage egg hatch, the percentage of mobile nematodes trended higher for RKN + Bb, than for RKN alone, but the difference was not significant. The percentage of immobile nematodes increased over time for both trials (Fig. 6). However, when compared between treatments, immobile nematodes (%) was greater in RKN alone over time in trial 1, but RKN + Bb treatment was greater than RKN alone in trial 2. However, no significance was recorded in both the trials.

The presence of Bb in the RKN egg suspension extracted from tomato roots in the greenhouse experiment was confirmed by growth of the fungus on BSM following inoculation of 500 µl of egg suspension.

## Discussion

Results of the gnotobiotic assay confirmed that Bb was endophytic in ‘Rutgers’ tomato. This cultivar was included in the current study because ‘Rutgers’ is very susceptible to RKN. Colonization patterns of ‘Rutgers’ by Bb were similar to those of ‘Mountain Spring’, which has been used in other studies where endophytic Bb 11-98 was shown to reduce disease from the soilborne pathogen *Rhizoctonia solani* (Bishop, 1999; Ownley et al., 2004, 2008; Seth, 2001). Overall, the extent of colonization appeared to be greater in ‘Rutgers’ than ‘Mountain Spring’. Differences in endophyte colonization of specific plants or cultivars could be due to differences in plant genes related to resistance or susceptibility, presence of specific plant chemicals that enhance or inhibit the growth of Bb, or growth of other microorganisms that compete with the endophyte (Greenfield et al., 2016). More studies are needed on the preference of endophytes for their plant hosts.

Our research findings indicate that Bb colonization was higher in stems and leaves compared to roots in both tomato cultivars (Rutgers and Mountain Spring). Bb was applied as a seed treatment, enabling the fungus to colonize the radicle and hypocotyl as they emerged from the seed. From the hypocotyl, the fungus had an early opportunity to colonize cotyledons, stem, and true leaves, as well as roots developing from the radicle. Competition from microorganisms is likely greater for colonization of roots than colonization of above ground parts because the rhizosphere is a more hospitable environment (above ground plant parts are exposed to UV light and reduced water and nutrients). It has been suggested that fungal colonization is more likely to occur on plant parts where it was applied directly first, than to distant plant parts (Akello et al., 2007, 2009; Tefera and Vidal, 2009). Methods used to isolate endophytes to confirm their presence could also play a role in where they are found on or in the plant. Isolation of an endophyte from plant tissues distant from the place of plant inoculation (El-Deeb et al., 2012) shows that the endophyte can move from one part of the plant to another (Posada et al., 2007), and demonstrates a pattern of systemic colonization (Tefera and Vidal, 2009).

In this study, a systemic colonization pattern of tomato was noted with significant differences in percent colonization among plant tissues. These results agree with previous observations that Bb can establish as an endophyte throughout the entire plant, especially after seed treatment (Akutse et al., 2013; Ownley et al., 2008; Quesada-Moraga et al., 2009). Contrary to results of the current study, there are research reports (Wearn et al., 2012; Yan et al., 2015) that Bb does not systemically colonize some plants or plant parts. This may be related to specific Bb isolates used or plant tissue type (Wearn et al., 2012; Yan et al., 2015).

The differential colonization rate of Bb that we noted in tissues of the two tomato cultivars agree with Biswas et al. (2012), who demonstrated that when Bb was artificially applied to jute, percent colonization was higher in leaves (56%) compared to stems (13%) and capsules (42%). Similarly, Russo et al. (2015) showed that when Bb was inoculated by different methods, at different times, onto different crops, colonization rates varied. For example, the highest colonization rates were achieved with foliar spray with 100% of tobacco leaves at 7 days, 40% of wheat leaves at 14 days, 7.8% of corn seedling leaves at 7 days, and 24% of soybean seedling leaves at 7 days. Variation in the way that Bb colonizes various plant tissues could be related to its opportunistic endophytic fungal lifestyle (Landa et al., 2013; McKinnon et al., 2017; Vidal and Jaber, 2015). Further studies are needed to understand the nature of differential colonization patterns by Bb.

The lowest colonization by Bb was in roots in the current study, which contrasts with studies by Greenfield et al. (2016). They reported that soil drenches of Bb conidia around stem cuttings of cassava allowed Bb to successfully colonize cassava roots, but no colonization was noted in stems and leaves. Colonization was higher when plants were sampled 7 to 9 days after inoculation (84%) compared to 47‒49 days (40%) (Greenfield et al., 2016). McKinnon et al. (2018) inoculated corn seedlings with Bb by soaking roots in conidial suspensions, and reported that rhizosphere populations of Bb declined at 30 days after inoculation unless plants were subjected to intensive wounding of foliage to crudely simulate herbivory. McKinnon et al. (2018) suggested that this could be an adaptive strategy of the fungus to increase the potential encounter of susceptible insect hosts.

Researchers have reported antagonism of Bb to nematodes in vitro. Ekanayake and Jayasundara (1994) demonstrated that beauvericin, a secondary metabolite produced by Bb, had weak nematicidal activity against root-knot nematode. Chen et al. (1996) found that Bb showed less nematode egg parasitism than other fungi, but Sun et al. (2006) conducted an in vitro assay and reported that Bb parasitized 100% of eggs of *Meloidogyne hapla* (northern root-knot nematode) and delayed egg hatching by 36% and caused 18.1% juvenile mortality. Liu et al. (2008) conducted an in vitro assay to investigate the effect of Bb culture filtrate against eggs and juveniles of *M. hapla*. They reported that cultural filtrate of *B. bassiana* inhibited *M. hapla* juveniles by 99% compared to the chemical pesticide Aldicarb that caused 89% inhibition. Zhao et al. (2013) evaluated eight isolates (Snef2607, Snef2615, Snef2636, Snef2637, Snef2568, Snef2598, Snef2626, and Snef2601) of Bb applied as culture filtrates on four nematodes: *M. incognita* (second-stage juveniles – J2), *Heterodera glycines* (J2), *Aphelenchoides besseyi*, and *Caenorhabditis* spp. They reported differential Bb toxicity to each nematode species. The Bb isolates caused high mortality of *M. incognita* (J2), *H. glycines* (J2), and *Caenorhabditis* spp., but were less effective against *Aphelenchoides besseyi*. Although other studies have reported nematicidal activity of Bb, this was not supported by results of the present study, even though we have established that the Bb isolate we used can produce beauvericin in culture (Leckie et al., 2008). The difference in results could be related to methodology, characteristics of other Bb isolates studied, or differences in tomato cultivars.

We investigated whether endophytic Bb has any antagonistic effect on RKN in a tomato under greenhouse conditions. Based on results, root galling and egg number of RKN per root increased with endophytic Bb on tomato plants, compared to endophyte-free control plants.

Data on various measurements of growth and plant development indicated that there were few differences between Bb-treated and endophyte-free plants when challenged with RKN. At 60 DAT, fresh root weight was an exception. Roots treated with Bb and inoculated with RKN were consistently larger than controls (no Bb or RKN) or roots with Bb and were larger than (Trial 1) or the same as (Trial 2) RKN only. Extensive galling was likely the cause of increased mass of roots with Bb.

Our data on root galling and egg counts per root were more direct measures of RKN success on plants (or lack of inhibition by Bb). More root galling and higher egg counts/root were recorded for the RKN + Bb treatment than for RKN alone, and this difference was significant in one trial. Mwaura et al. (2017) evaluated Bb by soil inoculation together with the potato tuber rot nematode (*Ditylenchus destructor*) and the stem and bulb nematode (*D. dipsaci*) in two greenhouse experiments. Our results agree with Mwaura et al. (2017), who reported that the combination of Bb with these two nematode species reduced potato tuber weight and yield and resulted in higher nematode population densities.

Our results suggest that endophytic Bb isolate 11-98 is not a good candidate for biocontrol of RKN in a susceptible tomato cultivar, such as ‘Rutgers’, and raise several questions, such as, Does Bb have triggering factors that increase or aggravate RKN root galling and egg production in ‘Rutgers’ tomato? Does ‘Rutgers’ have any triggering genes in response to Bb infection that might increase nematode infection? Are our results specific for the cultivar studied? Additional research is needed to answer these questions.

In the in vitro assay, using eggs extracted from greenhouse nematode treatments (RKN and RKN + Bb), with no additional Bb, there was a trend toward increased egg hatch with Bb + RKN, compared to RKN alone, but the differences were not significant. Conversely, there were higher percentages of mobile nematodes with RKN + Bb than RKN alone, but differences were not significant. There were also no significant differences in the daily percentages of immobile nematodes between the two treatments, in fact, there were no similar patterns between the two trials.

Our results are contrary to those of Liu et al. (2008), who conducted in vitro and in vivo studies on the effect of Bb on *M. hapla*. They demonstrated a nematicidal effect against *M. hapla* when Bb was applied as a ‘culture filtrate’ soil drench in tomato plants. The culture filtrate of Bb reduced egg hatch, increased nematode mortality in vitro, and reduced nematode populations in soil. Treatment with Bb also reduced gall formation and production of egg masses. Similarly, Kepenekci et al. (2017) found that Bb was effective on *Meloidogyne* and demonstrated that when Bb (isolates F-56 and F-63) was applied as conidial suspensions (10^6^, 10^7^, and 10^8^ CFU/ml), four times at different plant growth stages (15 days before planting, at planting, and 15 and 30 days after planting) on *M. incognita* and *M. javanica*-infected tomato under greenhouse conditions, Bb had high nematicidal activity and reduced root galling, as well as increased crop yield. The highest concentration of Bb (log 8/ml) had the greatest inhibitory effect.

In conclusion, Bb 11-98 was not effective in reducing root-knot nematode activity, including egg hatch and root galling, in ‘Rutgers’ tomato. The reason for the lack of effectiveness of Bb 11-98 is not clear. The endophyte was applied as a seed treatment and soil drench. In addition, Bb appeared to increase root galling and egg count in greenhouse tests. Although isolates of Bb have been reported as effective biocontrol agents against *Meloidogyne* spp. and other plant parasitic nematodes, it was not the case in this greenhouse and laboratory research. Endophytic Bb isolate 11-98 is not a good candidate for biocontrol of RKN in a susceptible tomato cultivar, such as ‘Rutgers’.
